# Efficacy and safety of Gantong Granules in the treatment of common cold with wind-heat syndrome: study protocol for a randomized controlled trial

**DOI:** 10.1186/s13063-015-0735-9

**Published:** 2015-05-19

**Authors:** Jie Min, Xiao-qiang Li, Bin She, Yan Chen, Bing Mao

**Affiliations:** Department of Integrated Traditional and Western Medicine, West China Hospital of Sichuan University, 37 Guoxue Lane, Chengdu, 610041 Sichuan Province People’s Republic of China; Anesthesia Department, West China Hospital of Sichuan University, 37 Guoxue Lane, Chengdu, 610041 Sichuan Province People’s Republic of China

**Keywords:** Common cold, Wind-heat syndrome, Gantong Granules, Randomized controlled trial, Traditional Chinese medicine

## Abstract

**Background:**

Although the common cold is generally mild and self-limiting, it is a leading cause of consultations with doctors and missed days from school and work. In light of its favorable effects of relieving symptoms and minimal side-effects, Traditional Chinese Medicine (TCM) has been widely used to treat the common cold. However, there is a lack of robust evidence to support the clinical utility of such a treatment. This study is designed to evaluate the efficacy and safety of Gantong Granules compared with placebo in patients with the common cold with wind-heat syndrome (CCWHS).

**Methods/Design:**

This is a multicenter, phase IIb, double-blind, placebo-controlled and randomized clinical trial. A total of 240 patients will be recruited, from 5 centers across China and randomly assigned to the high-dose group, medium-dose group, low-dose group or placebo control group in a 1:1:1:1 ratio. All subjects will receive the treatment for 3 to 5 days, followed by a 7-day follow-up period. The primary outcome is the duration of all symptoms. Secondary outcomes include the duration of primary symptoms and each symptom, time to fever relief and time to fever clearance, change in TCM symptom score, and change in Symptom and Sign Score.

**Discussion:**

This trial will provide high-quality evidence on the efficacy and safety of Gantong Granules in treating CCWHS, and help to optimize the dose selection for a phase III clinical trial.

**Trial registration:**

The registration number is ChiCTR-TRC-14004255, which was assigned by the Chinese Clinical Trial Registry on 12 February 2014.

## Background

The common cold has been with humanity since antiquity. It is the most widespread illness in humans with the average adult getting two to four colds a year and the average child getting six to eight colds [1]. Although the common cold is generally mild and self-limiting, it is the leading cause of consultations with doctors and days missed annually from school and work [2]. There are more than 200 viruses associated with the common cold and the rhinovirus is the most common cause [3]. The symptoms of the common cold can vary considerably between different viruses and often include a tickle or sore throat, sneezing, runny nose, nasal congestion, coughing, mild fever and general malaise [1,4]. So far, there is no proven intervention for treating or preventing the common cold. Thus, current treatments mainly focus on symptom relief. Few available therapies, including zinc, vitamin C, *Echinacea*, garlic, antihistamines, intranasal ipratropium, corticosteroids, antibiotics, and so on [5-14], are recommended due to ineffectiveness, questionable benefit, or adverse effects. Traditional Chinese Medicine (TCM) is a unique and well-established system of medicine that has benefited numerous people worldwide. In fact, in China and many other countries, a number of patients prefer TCM treatment rather than Western medicine to treat the common cold. As the most important component of TCM, Chinese medicinal herbs are mainly derived from plants and usually incorporate one or more herbs to treat a disease. Based on TCM syndrome differentiation, the common cold can be divided into the wind-cold, wind-heat or summer-heat dampness syndrome according to its symptoms and TCM signs, such as fever, headache, fatigue, aversion to cold, sneezing, nasal congestion, nasal discharge, nausea, vomiting, tongue proper, tongue coating and condition of pulse [15]. The common cold with wind-heat syndrome (CCWHS) is the most common type and occurs in all seasons; it is primarily characterized by fever, mild aversion to cold, sore throat, nasal congestion and discharge. According to the treatment principles of TCM, CCWHS should be treated by clearing heat and releasing stagnated *Lung-Qi*.

Gantong Granules is a novel Chinese patent medicine that is manufactured by Beikelian Pharmaceutical & Technology Co., Ltd, Shenzhen, China. It is mainly composed of *Chaihu* (*Radix bupleuri*), *Gegen* (*Radix puerariae*), *Niubangzi* (*Fructus arctii*), *Bohe* (*Herba menthae*), *Banlangen* (*Radix isatidis*), *Chuanbeimu* (*Bulbus fritillariae unibracteatae*), *Qianhu* (*Radix peucedani*) and *Jiegeng* (*Radix platycodi*). All of these ingredients have been approved by the China Food and Drug Administration (CFDA). Gantong Granules has been proven effective in treating CCWHS based on clinical experiences in the Affiliated Hospital of Changchun College of TCM. Preclinical pharmacologic experiments showed that Gantong Granules had the effects of clearing heat, relieving pain, reducing inflammation and alleviating cough. A second-stage clinical trial also demonstrated that it could improve symptoms of CCWHS. In addition, no evidence of an adverse or toxic effect has been found in toxicological studies.

In accordance with the Drug Administration Law of the People’s Republic of China and *Good Clinical Practice* (GCP) issued by CFDA, this phase IIb clinical trial is well-designed to evaluate the efficacy and safety of Gantong Granules compared with placebo in patients with CCWHS. Additionally, it is expected to find a dose-effect relationship and provide useful evidence for a Phase III clinical program.

## Methods/Design

### Design

**Figure 1:**
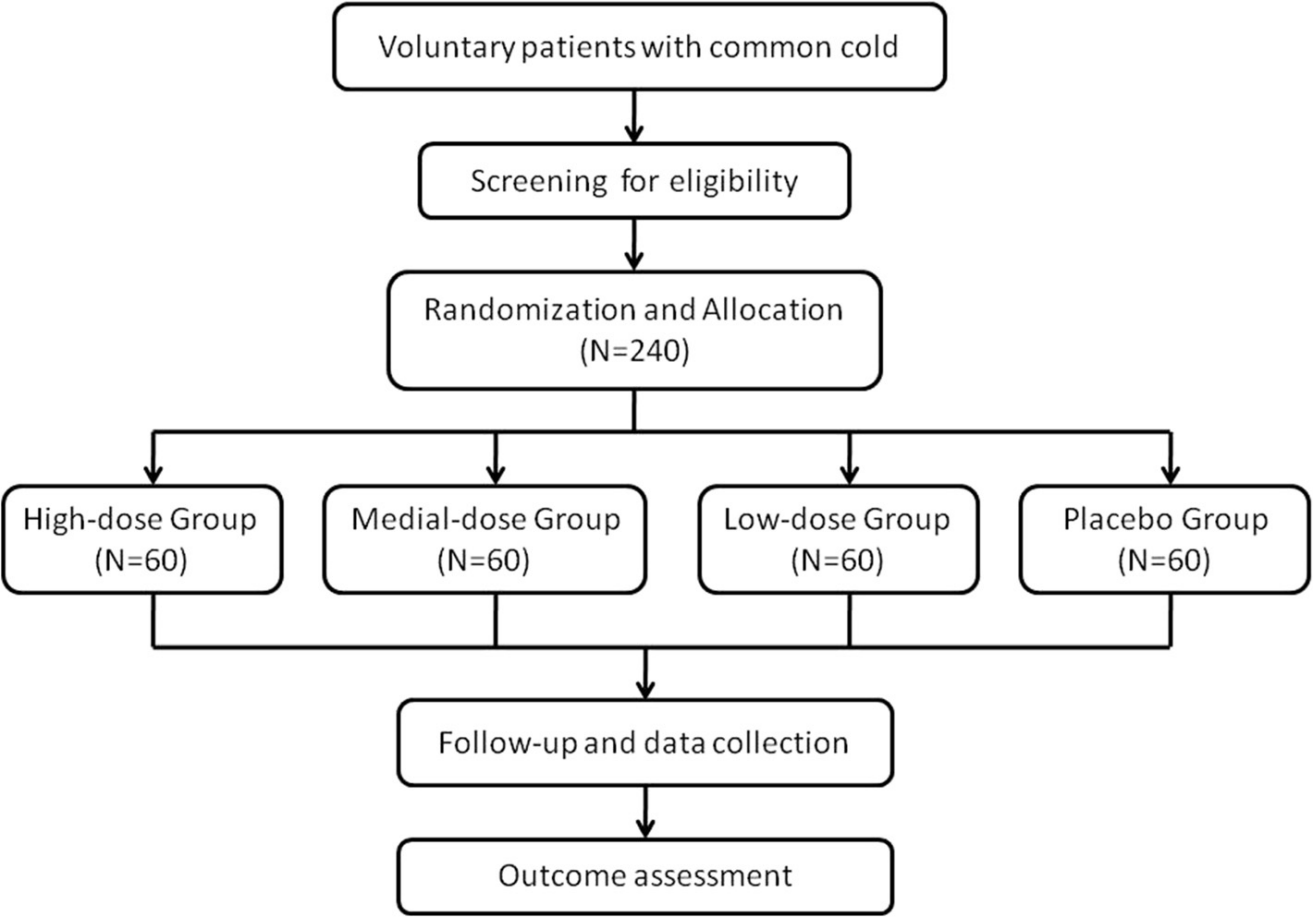
Study flow chart.

This study is a multicenter, phase IIb, double-blind, placebo-controlled and randomized clinical trial. This trial has been authorized by the CFDA (Approval Number 2004 L04586) and registered with the Chinese Clinical Trial Registry (ChiCTR-TRC-14004255). In addition, the protocol is conducted in accordance with the *Declaration of Helsinki* (2008) and the *Good Clinical Practice* G*uidelines* [16]. The study is financially supported by Beikelian Pharmaceutical & Technology Co., Ltd, Shenzhen, China, which had or will have no role in the study design, analysis, data interpretation or decision to submit results. Across the five trial centers, all investigators are appropriately qualified by training and experiences to conduct and supervise the trial. All patients have to provide their written informed consent prior to study entry. The flow chart of this study is shown in Figure 1.

### Ethics

The trial protocol has been approved by five local ethics committees, including the Clinical Trials and Biomedical Ethics Committee of West China Hospital of Sichuan University (Number TCM-2013-08), the Clinical Trials and Biomedical Ethics Committee of the Affiliated Hospital of Chengdu University of TCM, the Clinical Trials and Biomedical Ethics Committee of the First Hospital of Hunan University of TCM, the Clinical Trials and Biomedical Ethics Committee of the Affiliated Hospital of Nanjing University of TCM, and the Clinical Trials and Biomedical Ethics Committee of the Affiliated Hospital of Jangxi University of TCM. Research assistants will introduce the trial to potential subjects, and give them information sheets about the trial. All eligible patients have to give their signed informed consent before enrollment. The privacy of all participants will be protected. Personal medical records will be taken by investigators and inspectors, who will promise to keep that secret. Regulatory authorities have the right to examine the test data. Data anonymity will be used in process of data management. Finally, the data of all subjects will be kept centrally.

### Recruitment

We plan to recruit patients through advertisements and recommendations. A total of 240 patients will be recruited in this trial at the following 5 hospitals across China: 1) West China Hospital of Sichuan University, 2) the Affiliated Hospital of Chengdu University of TCM, 3) The First Hospital of Hunan University of TCM, 4) the Affiliated Hospital of Nanjing University of TCM, 5) the Affiliated Hospital of Jangxi University of TCM. Forty-eight patients will be recruited at each center.

### Randomization and blinding

Using a stratified block randomization method, 240 eligible patients will be randomly allocated to the high-dose group, medium-dose group, low-dose group or placebo control group in a 1:1:1:1 ratio. The randomization sequence will be generated using the PRCO PLAN function of the analysis system of SAS software (SAS, Cary, NC, USA). The randomization lists will be concealed in a lightproof sealed envelope. The sealed envelopes will be kept by the leader and the sponsor of the study. Treatment allocation will be blinded to the participants and investigators throughout the course of the study. The emergency envelope will be saved in each center, and only be opened for medical emergency purposes.

### Diagnostic criteria for the common cold

The diagnosis in Western medicine is established according to *Practice of Internal Medicine* (2009, Version 13) [17] and *Consensus on the Diagnosis and Treatment of Common Cold by Respiratory Branch of Chinese Medical Association and Emergency Branch of Chinese Medical Association* [18]. Patients should have typical clinical manifestations of the common cold, such as sneezing, nasal discharge, nasal congestion, and/or sore throat, as well as excluding other diseases.

The TCM diagnosis of CCWHS is based on the *Guidelines for Clinical Research of New Chinese Medicine* [19]. The TCM diagnostic criteria for CCWHS are listed in Table 1. To be diagnosed with CCWHS, patients should have all the primary symptoms and at least three of the secondary symptoms, as well as the TCM signs for the tongue and pulse.

**Table 1:** **Traditional Chinese Medicine (TCM) diagnostic criteria**

**Category**	**Symptoms and signs**
Primary symptoms	Fever, mild aversion to cold, sore throat
Secondary symptoms	Sore limbs, nasal congestion, nasal discharge, thirsty, cough
Signs for the tongue	Red tongue and thin yellow tongue coating
Signs for the pulse	Floating and rapid pulse

### Inclusion criteria

- Diagnosis of common cold according to Western medicine
- Diagnosis of CCWHS according to TCM
- Aged between 18 and 65 years
- Presenting within 48 hours after symptom onset
- Willing to participate and sign the informed consent

### Exclusion criteria

- Patients with influenza, acute bacterial sinusitis, allergic rhinitis, streptococcal pharyngitis and herpangina
- Patients with imaging manifestations of pulmonary infection
- Patients with serious primary diseases of the cardiovascular, pulmonary, kidney, and hematological system
- Patients with liver function levels (such as alanine aminotransferase (ALT), aspartate aminotransferase (AST), serum total bilirubin (STB), alkaline phosphatase (ALP), and/orγ-glutamyl transpeptidase (γ-GT)) 1.5 times higher than the upper limit of normal, abnormal serum creatinine, positive urine protein qualitative test, white blood cell count < 3.0 × 10^9^/L or > 10.0 × 10^9^/L, and/or neutrophil percentage > 80%.
- Patients who are taking any medication to relieve symptoms
- Patients with a body temperature higher than 38.5°C
- Pregnant women, lactating women, or women who have a birth plan
- Patients with an allergic constitution or being allergic to the study drug
- Mentally or legally disabled patients
- Patients who are participating in or have participated in another drug clinical trial within the last 3 months
- Patients who have been judged by the investigator as inappropriate to participate in the clinical trial.

### Rejection criteria

- Misdiagnosis
- Not taking any study drug during the trial
- Using forbidden drugs or treatments during the trial
- No evaluable records after drug administration

### Withdrawal criteria

- Worsening conditions during the trial, including a body temperature higher than 39.0°C
- Experiencing anaphylaxis or serious adverse events during the trial
- Quitting the clinical trial voluntarily
- The study drug is not taken as directed, or is taken at doses <80% or >120% of the requirement

### The study will be suspended early or terminated if any of the following occur:

- Serious adverse event (SAE)
- The efficacy of the test drug is found to be poor or even ineffective
- Flawed protocol or significant deviation from the well-designed protocol
- The pharmaceutical supervisory and administrative department decides to terminate the study for any reason
- The sponsor decides to terminate the trial due to management or funding problems

### Interventions

**Figure 2:**
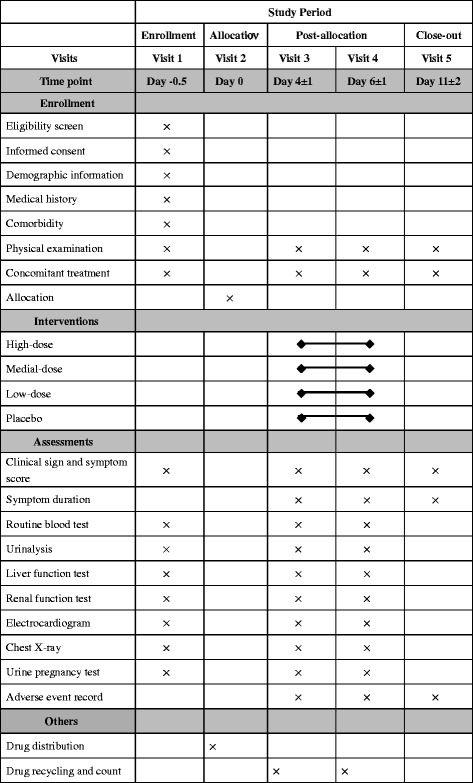
Study schedule for patients.

Gantong Granules and placebo are provided and manufactured by Beikelian Pharmaceutical & Technology Co., Ltd, Shenzhen, China. Considering the characteristics of Chinese herbal medicine, placebo is prepared by 10-fold dilution of 5 g of Gantong Granules and then condensed into granules. The minimum effective dose of Gantong Granules is 5 g. After 10-fold dilution, the placebo will not have any clinical or pharmacological effects. Placebo is almost identical to the test drug in appearance, smell and taste. This preparation of placebo has been used in herbal medicine trials in China and Japan. All drugs are concealed in uniform packages with the same labels and each package contains 5 plus 1 days’ dosage. Patients in the high-dose group, medium-dose group, low-dose group or placebo control group, will receive Gantong Granules 15 g, Gantong Granules 10 g plus placebo 5 g, Gantong Granules 5 g plus placebo 10 g or placebo 15 g, respectively. All drugs will be dissolved in 200 ml warm water and taken orally before each meal 3 times daily for 3 to 5 days. If all symptoms disappear within 3 days, the study drugs should be taken for just 3 days; if not, for 5 days. During the study, patients will be visited five times by the investigators. The follow-up visits last for 7 days after the treatment. Details of study procedures are shown in Figure 2.

### Concomitant treatments and forbidden drugs

Patients will be allowed to continue using medications for comorbidities, such as hypertension or diabetes. The dosage, duration and name of any concomitant medication or treatment must be recorded carefully in the case report form (CRF). When the body temperature of a participant rises to higher than 39.0°C, physical cooling and/or paracetamol can be used to reduce the fever. Any other Western or Chinese therapy or medication that may affect the study results is prohibited during the trial.

### Outcome measures

#### Primary outcomes

The primary outcome of the study is the duration of all symptoms, which is defined as the number of hours from study enrollment to the time when the patient’s illness symptoms completely resolve. Each participant will be instructed to record any changes in symptoms in the patient diary, in which the data can be acquired. Investigator will make an assessment of the duration based on these data.

#### Secondary outcomes

Secondary outcomes include duration of primary symptoms and each symptom, time to fever relief and time to fever clearance, change in TCM symptom score, and change in Symptom and Sign Score.

### Time to fever relief and time to fever clearance

In this study, all patients will be asked to record their axillary temperature in the patient diary every 2 hours within the first 24 hours; after that, if the temperature is equal to or higher than 37.3°C, it should be recorded every 4 hours, and if the temperature is lower than 37.3°C, then it should be recorded at 8 am. and 4 pm. every day. If the temperature is below 37.3°C and no longer rises for 48 hours, subjects do not need to continue recording. Time to fever relief is defined as the time from the first dose of the study drug until the body temperature drops 0.5°C or to normal; time to fever clearance is defined as the time from the first dose of the study drug until the body temperature drops below 37.3°C and no longer rises for 48 hours(calculated as the time to the start of the 48-hour period).

### Change in TCM symptom score

**Figure 3:**
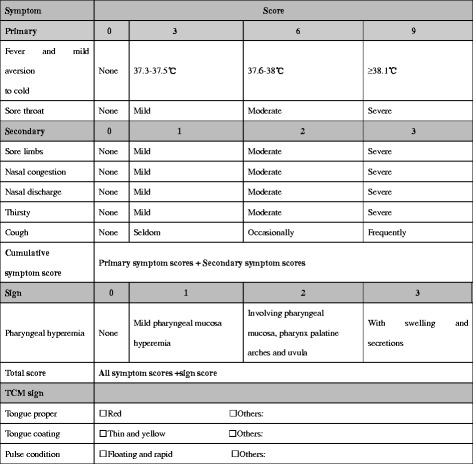
Symptom and Sign Scoring system.

The TCM symptom score system used in the study follows the *Guidelines for Clinical Research of New Chinese Medicine* [19], in which all symptoms are given graded scores (Figure 3). TCM signs will also be assessed, but not scored. The sum of all symptom scores is the cumulative TCM symptom score. The change in cumulative TCM symptom score is assessed by the percentage of symptom score reduction (PSSR), which is calculated according to the following formula:

$$ \mathrm{PSSR}\kern0.5em =\kern0.6em \left(\frac{symptom\  score\  before\  treatment\hbox{-} symptom\  score\  after\  treatment}{symptom\  score\  before\  treatment}\right)\kern0.5em \times \kern0.5em 100\% $$

### Change in Symptom and Sign Score

Pharyngeal hyperemia will be given graded scores as common cold sign (Figure 3). The change in cumulative Symptom and Sign Score is evaluated by the percentage of Symptom and Sign Score reduction (PSSSR), which is calculated according to the following formula:

$$ \mathrm{PSSSR}\kern0.5em =\kern0.5em \left(\frac{symptom\  and\  sign\  score\  before\  treatment\hbox{-} symptom\  and\  sign\  score\  after\  treatment}{symptom\  and\  sign\  score\  before\  treatment}\right)\kern0.5em \times \kern0.5em 100\% $$

### Safety outcomes

Physical examination and some laboratory tests will be performed both before and after treatment for safety assessment. Physical examination includes temperature, respiration, blood pressure, heart rate and weight. Laboratory tests include routine blood test, urine analysis, liver function test (ALT, AST, ALP, STB, and γ-GT), renal function test (serum creatinine, microalbuminuria, and serum cystatin C), and electrocardiogram. Beyond that, before treatment, all subjects need to undergo a chest X-ray, and female patients of childbearing age need to take a urine pregnancy test.

### Adverse event reporting

It is very important to ensure all patients’ safety during the trial. Adverse events (AEs) should be recorded in detail on the adverse event form (AEF). All patients experiencing an AE will be treated appropriately. Regarding a SAE, the investigator must record it on the SAE form, and it should be reported to the principal investigator, CFDA, ethics committees and the sponsor within 24 hours.

### Quality control

All investigators are appropriately qualified by training and experience to conduct and supervise the trial. The principal investigator in each center will be responsible for the implementation of the clinical trial which should be in compliance with standard operation procedure. Throughout this trial, specialized quality inspectors will regularly review and monitor the experimental data from CRFs every week. Monitoring results should be presented to the principal investigator. The inspector will protect the privacy of the participants.

### Data management

After review by the investigators and inspectors, completed CRFs will be sent to a specified statistics center, where data entry and management will be completed by two independent data administrators to ensure data accuracy. All data will be managed by DAS (Direct Attached Storage) for electronic data management system. To confirm the database, the principal investigator, the sponsor and statisticians will perform a blind review, and make a statistical analysis plan. Finally, the data will be locked and analyzed in accordance with the statistical analysis plan.

### Sample size calculation

Based on a previous clinical study [20], the mean duration of all symptoms for the common cold was estimated to be 7 days with a standard deviation of 3 days. Reduction in the duration of all symptoms by 2 days is supposed to be clinically relevant. Using Package for Encyclopedia Medical Statistics (PEMS) 3.2 with 90% power and α = 0.05 (2-sided), the sample size needs to be 48 cases for each group. Considering a drop-out of 20%, 60 subjects should be recruited in each group. Consequently, the total sample size is determined to be 240 patients.

### Statistical analysis

The data analysis will be performed by professional statisticians, in accordance with the statistical analysis plan for this trial. The full analysis set (FAS) is the primary analysis set for efficacy with an intention-to-treat (ITT) principle. In the FAS, all patients will be treated with at least one dose of the study drug and recorded with at least one clinical observation in the study. All subjects without any major protocol deviations will be involved in per-protocol set (PPS). The safety analysis will be conducted for randomized subjects who have completed at least one study visit and have safety data. Descriptive statistics will be performed on continuous variables, frequencies and categorical variables. All symptom duration, primary symptom duration, time to fever relief and time to fever clearance will be estimated by the Kaplan-Meier technique and be compared by the stratified log-rank test. Comparisons among groups will be conducted by an analysis of variance (ANOVA) and least significant difference*t*-test. In this multicenter trial, the Cochran-Mantel-Haenszel test, stratified by clinical centers, and the logistic regression model, adjusted for covariates, will be used. All data will be processed by a professional statistician using SAS 9.2 software (SAS, Cary, NC, USA), and a 2-sided *P*-value of < 0.05 is considered to be statistically significant.

## Discussion

The common cold is something that one may experience multiple times throughout his or her lifetime. To date, however, there is no well-proven treatment or cure for this disease. It is widely recognized that the randomized controlled trial is a ‘gold standard’ methodology for evaluating the clinical efficacy and safety of an intervention. Although Chinese herbs have been widely used to treat the common cold in China and many other countries, the quality of most prior studies was assessed to be generally low due to methodological limitations like inadequate randomization, lack of double blinding, non-placebo control, incomplete outcome data, and so on [21,22]. So, there is a lack of robust evidence to support the clinical utility of TCM treatment.

For the above reasons, this protocol is rigorously-designed in accordance with the CONSORT (Consolidated Standards of Reporting Trials) statement and SPIRIT (Standard Protocol Items: Recommendations for Interventional Trials) statement [23,24]. As far as we know, this study will be the first multicenter, double-blind, placebo-controlled and randomized clinical trial that is designed to treat CCWHS. Based on scientific and objective assessment, this study will provide high-quality evidence on the efficacy and safety of Gantong Granules in treating CCWHS, and may help to optimize the dose selection for a phase III clinical trial.

## Trial status

The study is currently in the process of recruiting participants in the five trial centers.

## Abbreviations

AEs: adverse events
AEF: adverse event form
ALP: alkaline phosphatase
ALT: alanine aminotransferase
ANOVA: analysis of variance
AST: aspartate aminotransferase
CCWHS: common cold with wind-heat syndrome
CFDA: China Food and Drug Administration
CONSORT: Consolidated Standards of Reporting Trials
CRF: case report form
DAS: Direct Attached Storage
FAS: full analysis set
γ-GT: γ-glutamyl transpeptidase
GCP: Good Clinical Practice
ITT: intention-to-treat
PEMS: Package for Encyclopedia Medical Statistics
PPS: per-protocol set
PSSR: percentage of Symptom Score reduction
PSSSR: percentage of Symptom and Sign Score reduction
SAE: severe adverse events
SPIRIT: Standard Protocol Items: Recommendations for Interventional Trials
STB: serum total bilirubin
TCM: Traditional Chinese Medicine

## References

[1] Heikkinen T, Jarvinen A (2003). The common cold. Lancet.

[2] Fendrick AM, Monto AS, Nightengale B, Sarnes M (2003). The economic burden of non-influenza-related viral respiratory tract infection in the United States. Arch Intern Med.

[3] Eccles R (2005). Understanding the symptoms of the common cold and influenza. Lancet Infect Dis.

[4] Roxas M, Jurenka J (2007). Colds and influenza: a review of diagnosis and conventional, botanical, and nutritional considerations. Altern Med Rev.

[5] Murdoch DR, Slow S, Chambers ST, Jennings LC, Stewart AW, Priest PC (2012). Effect of vitamin D3 supplementation on upper respiratory tract infections in healthy adults: the VIDARIS randomized controlled trial. JAMA.

[6] Singh M (2013). Heated, humidified air for the common cold. Cochrane Database Syst Rev.

[7] Singh M, Das RR (2013). Zinc for the common cold. Cochrane Database Syst Rev.

[8] AlBalawi ZH, Othman SS, AlFaleh K (2013). Intranasal ipratropium bromide for the common cold. Cochrane Database Syst Rev.

[9] Lissiman E, Bhasale AL, Cohen M (2014). Garlic for the common cold. Cochrane Database Syst Rev.

[10] De Sutter AI, van Driel ML, Kumar AA, Lesslar O, Skrt A (2012). Oral antihistamine-decongestant-analgesic combinations for the common cold. Cochrane Database Syst Rev.

[11] Douglas RM, Hemilä H, Chalker E, Treacy B (2007). Vitamin C for preventing and treating the common cold. Cochrane Database Syst Rev.

[12] Linde K, Barrett B, Wölkart K, Bauer R, Melchart D (2006). Echinacea for preventing and treating the common cold. Cochrane Database Syst Rev.

[13] Kenealy T, Arroll B (2013). Antibiotics for the common cold and acute purulent rhinitis. Cochrane Database Syst Rev.

[14] Hayward G, Thompson MJ, Perera R, Del Mar CB, Glasziou PP, Heneghan CJ (2012). Corticosteroids for the common cold. Cochrane Database Syst Rev.

[15] Jiao Y, Liu J, Jiang L, Liu Q, Li X, Zhang S (2013). Guidelines on common cold for Traditional Chinese Medicine based on pattern differentiation. J Tradit Chin Med.

[16] Vijayananthan A, Nawawi O (2008). The importance of Good Clinical Practice guidelines and its role in clinical trials. Biomed Imaging Interv J.

[17] Chen HZ (2009). Practice of internal medicine.

[18] Respiratory Physician Branch of Chinese Medical Association, Emergency Physician Branch of Chinese Medical Association (2012). Consensus on the diagnosis and treatment of common cold. Chin J Intern Med.

[19] Zhen XY (2002). Guidelines for clinical research of new Chinese medicine.

[20] Barrett B, Brown R, Rakel D, Mundt M, Bone K, Barlow S (2010). Echinacea for treating the common cold: a randomized controlled trial. Ann Intern Med.

[21] Zhong YQ, Fu JJ, Liu XM, Diao X, Mao B, Fan T (2010). The reporting quality, scientific rigor, and ethics of randomized placebocontrolled trials of traditional Chinese medicine compound formulations and the differences between Chinese and non-Chinese trials. Curr Ther Res Clin E.

[22] Wu T, Zhang J, Qiu Y, Xie L, Liu GJ (2007). Chinese medicinal herbs for the common cold. Cochrane Database Syst Rev.

[23] Kenneth FS, Douglas GA, David M (2010). CONSORT 2010 statement: updated guidelines for reporting parallel group randomized trials. Ann Intern Med.

[24] Chan AW, Tetzlaff JM, Altman DG, Laupacis A, Gøtzsche PC, Krleža-Jerić K (2013). SPIRIT 2013 statement: defining standard protocol items for clinical trials. Ann Intern Med.

